# Effect of an Immersive Virtual Reality Intervention on Pain and Anxiety Associated With Peripheral Intravenous Catheter Placement in the Pediatric Setting: A Randomized Clinical Trial

**DOI:** 10.1001/jamanetworkopen.2021.22569

**Published:** 2021-08-25

**Authors:** Jeffrey I. Gold, Michelle SooHoo, Andrea M. Laikin, Arianna S. Lane, Margaret J. Klein

**Affiliations:** 1 Department of Anesthesiology, Keck School of Medicine, University of Southern California, Los Angeles; 2 University Center of Excellence in Developmental Disabilities, University of Southern California, Los Angeles; 3 Department of Anesthesiology Critical Care Medicine, Saban Research Institute at Children’s Hospital Los Angeles, Los Angeles, California; 4 Department of Pediatrics, Keck School of Medicine, University of Southern California, Los Angeles; 5 Department of Psychiatry and Behavioral Sciences, Keck School of Medicine, University of Southern California, Los Angeles

## Abstract

**Importance:**

The inclusion of digital therapeutics (eg, virtual reality [VR] systems) for the management of pain and anxiety associated with routine acutely painful medical procedures may have a substantial impact on treatment adherence and improve long-term health outcomes among young patients.

**Objective:**

To determine whether a VR intervention decreases pain and anxiety among patients undergoing peripheral intravenous catheter (PIVC) placement compared with standard care in the pediatric setting.

**Design, Setting, and Participants:**

This randomized clinical trial was conducted from April 12, 2017, to July 24, 2019, among 107 patients aged 10 to 21 years who were undergoing PIVC placement in 2 clinical settings (a radiology department and an infusion center) at an urban pediatric academic medical center in the US. Patients, caregivers, and clinicians completed pre-PIVC and post-PIVC placement questionnaires measuring patient pain, anxiety, and anxiety sensitivity; only participants with complete data from before and after PIVC placment were included in the analyses.

**Interventions:**

Patients were randomized to receive standard care (simple distraction techniques [eg, music, coloring, singing, and talking] and the application of numbing cream) or a VR intervention using a balanced computer-generated randomization scheme stratified by sex. All patients who received the VR intervention were offered concurrent standard care; however, VR plus standard care was not specifically examined.

**Main Outcomes and Measures:**

Primary outcomes were patient pain (measured by the Faces Pain Scale–Revised) and anxiety (measured by a visual analogue scale) reported by the patient, caregiver, and clinician after PIVC placement. Outcomes were analyzed using generalized linear modeling with backward stepwise selection for final model building.

**Results:**

A total of 107 patients (median age, 14.7 years [interquartile range, 12.8-16.9 years]; 63 male participants [58.9%]) completed the clinical trial; 54 patients received standard care, and 53 patients also received the VR intervention. Patients who received the VR intervention compared with standard care had significantly lower mean post-PIVC anxiety scores when patient-reported (1.85 points [95% CI, 1.28-2.41 points] vs 3.14 points [95% CI, 2.59-3.68 points]; *P* < .001) and clinician-reported (2.04 points [95% CI, 1.37-2.71 points] vs 3.34 points [95% CI, 2.69-3.99 points]; *P* = .002). Patients in the VR group vs the standard care group also had significantly lower mean post-PIVC pain scores when patient-reported (1.34 points [95% CI, 0.63-2.05 points] vs 2.54 points [95% CI, 1.78-3.30 points]; *P* = .002), caregiver-reported (1.87 points [95% CI, 0.99-2.76 points] vs 3.01 points [95% CI. 1.98-4.03 points]; *P* = .04), and clinician-reported (2.05 points [95% CI, 1.47-2.63 points] vs 3.59 points [95% CI, 2.97-4.22 points]; *P* < .001). Aside from lower levels of baseline pain and anxiety, no demographic variables among patients in the VR group were associated with lower levels of post-PIVC pain and anxiety.

**Conclusions and Relevance:**

In this randomized clinical trial, patients undergoing PIVC placement who received a VR intervention experienced significantly less anxiety and pain compared with those who received standard care. The use of patient, caregiver, and clinician data provided a variety of subjective information, as well as observable and objective data regarding perceived pain and anxiety beyond patient reporting alone.

**Trial Registration:**

ClinicalTrials.gov Identifier: CHLA-15-00549

## Key Points

**Question:**

Does a virtual reality (VR) intervention compared with standard care improve pain and anxiety outcomes among patients undergoing peripheral intravenous catheter (PIVC) placement in the pediatric setting?

**Findings:**

In this randomized clinical trial of 107 patients undergoing PIVC placement in 2 pediatric clinical settings plus their caregivers and clinicians, a VR intervention significantly reduced mean patient-reported post-PIVC anxiety and pain, caregiver-reported perceptions of patients’ pain, and clinician-reported perceptions of patients’ pain and anxiety.

**Meaning:**

In this study, a VR intervention significantly decreased pain and anxiety among patients undergoing PIVC placement in the pediatric setting.

## Teaser

This randomized clinical trial examines the effect of a virtual reality intervention vs standard care on pain and anxiety associated with peripheral intravenous catheter placement among patients receiving care in 2 pediatric clinical settings.

## Introduction

Immersive virtual reality (VR) technologies have continued to evolve over the past 20 years. The VR experience capitalizes on multisensory integration to transport the individual into computer-simulated worlds. It has also been asserted that immersive VR environments may provide more effective therapeutic interventions than standard practices (eg, bubbles, pinwheels, videos, and books) for the management of procedural pain and anxiety because of the combination of attentional demands and other cortical systems involved in VR gameplay.^1^

Investigators have experimented with advancements in commercial VR headsets (eg, Oculus Rift; Oculus VR) and mobile VR capabilities (eg, Samsung Gear VR; Samsung Electronics), leading to more affordable and feasible clinical trials examining the use of VR systems in health care settings.^2^ However, the heterogeneity of VR systems and environments has produced mixed scientific findings stemming from the inability of evidence-based evaluations to keep pace with continuously evolving technological innovations.^3,4^ Although there has been substantial expansion in the use of VR interventions in health care settings, the scientific evidence continues to lag behind advancements in technologies and software; relatively little is known regarding which patients may benefit from VR interventions and which procedures are best suited for incorporating VR systems.

Young patients who routinely undergo painful medical procedures, such as peripheral intravenous catheter (PIVC) placement or blood sample collection, often experience pain and distress, thereby increasing their risk of needle phobia, anxiety, and treatment nonadherence.^5^ This pain and distress reduces treatment satisfaction for the patient, caregiver, and clinician. Furthermore, routine procedures may induce adverse emotional or traumatic reactions, especially among patients with chronic conditions receiving medical treatment that may constitute frequent vascular access.^6^ Given the potential detriment of painful routine medical procedures for the patient, caregiver, and clinician, it is important to consider alternative strategies for the management of pain and anxiety beyond standard care. Standard care for the management of acute procedural pain in the pediatric setting includes simple distraction techniques (eg, music, coloring, singing, and talking) and the application of numbing cream.^7,8,9^ A 2008 survey of child life specialists found that VR intervention was one of the strategies used least frequently for pediatric pain management,^10^ even though a randomized clinical trial found that VR was an effective intervention for this purpose.^11^ An abbreviated Cochrane review similarly found that, across 28 randomized clinical trials of psychological interventions for pain management, nonpharmacological distraction interventions were among the most beneficial in supporting the reduction of pain and distress among children undergoing needle-related procedures.^12^

Virtual reality interventions have been reported to decrease pain during medical procedures, such as burn and wound care, port access, blood sample collection, intravenous (IV) placement and therapy, and dental procedures.^13,14,15,16,17,18,19,20^ These VR interventions can also be implemented within a health care setting at low cost^21^ and have been found to offer superior pain reduction for children compared with standard care during wound dressing changes^22^ and IV placements before computed tomographic scans.^17^

The use of VR systems has also been found to reduce reported symptoms of acute anxiety during medical procedures.^23^ Research suggests that negative emotions, such as anxiety, may facilitate pain, making anxiety an important area to consider for pain management.^24^ Gold et al^17^ found that pediatric patients receiving standard care during IV placement experienced a 4-fold increase in affective pain compared with patients receiving a VR intervention, highlighting the association between pain and anxiety among young patients undergoing routine medical procedures. Therefore, it is important to examine the association between acute pain and anxiety sensitivity, which refers to one’s propensity to be afraid of anxiety-related sensations.^5^

Research on the use of VR interventions has generally been conducted among small samples in 1 or 2 settings.^25,26,27^ Chan et al^25^ examined VR use in the emergency department and outpatient settings among children undergoing IV cannulation or venipuncture, reporting a statistically significant decrease in perceived pain among the VR group in the emergency department. However, in the pathology setting, both the VR and standard care groups experienced an increase in pain, with patients in the VR group reporting a lesser increase in pain than those in the standard care group. Previous research on VR interventions for managing procedural pain and anxiety in pediatric settings has been limited, comprising small samples and results that have mostly been reported in technological and nonpediatric publications, thereby limiting the impact or relevance of the findings for direct clinical services.

The current randomized clinical trial examined the effectiveness of a VR intervention compared with standard care for reducing patient pain and anxiety during PIVC placement in 2 pediatric clinical settings. Within the VR group, patient characteristics were examined to identify which patients would most benefit from the VR intervention.

## Methods

This study was approved by the institutional review board of Children’s Hospital Los Angeles. All patients provided written informed consent; for those younger than 18 years, patient assent and written informed consent from a parent or guardian were obtained. The study followed the Consolidated Standards of Reporting Trials (CONSORT) reporting guideline for randomized clinical trials. The trial protocol appears in Supplement 1.

### Study Population

The final study population included 107 patients aged 10 to 21 years, caregivers for patients younger than 18 years, and clinicians who conducted the PIVC procedure. Patients were scheduled to undergo PIVC placement in the department of radiology and imaging or the outpatient infusion center. All patients and caregivers spoke English or Spanish. Patients were excluded if they had a developmental delay, a history of seizure disorder, or visual or auditory deficits that would interfere with VR gameplay or if they had received pain or anxiety medication that morning.

### Procedures

This randomized clinical trial was conducted in 2 clinical environments (a radiology department and an infusion center) at an urban pediatric academic medical center (Children’s Hospital Los Angeles) from April 12, 2017, to July 24, 2019. Research staff (including A.S.L.) identified potentially eligible patients before their outpatient appointments and approached them in the waiting area to determine their interest and eligibility. Patients were enrolled after obtaining written informed consent. After enrollment, patients and their caregivers completed a battery of questionnaires via Qualtrics on tablet computers (iPad; Apple, Inc) to assess baseline patient pain and anxiety. Using a balanced computer-generated randomization scheme stratified by sex, patients were randomized to receive either standard care (standard care group) or a VR intervention (VR group). All patients in the VR group were offered concurrent standard care (eg, numbing cream); however, many patients in the group declined standard care, and VR plus standard care was not specifically examined. Study personnel were blinded to the patient’s treatment group until baseline measures were completed. Patients in both groups had access to standard care procedures for PIVC placement (ie, a topical numbing spray and the use of Buzzy Bee [Buzzy4Shots Australia and New Zealand], a vibrating device placed near the PIVC site for distraction from pain). For patients in the VR group, research staff briefly explained gameplay and fitted them with a VR headset. Patients began gameplay less than 5 minutes before their PIVC placement and concluded gameplay after successful vascular access. After PIVC placement, patients, caregivers, and clinicians rated patient pain and anxiety levels during the procedure.

### VR Equipment

Two mobile-based VR head-mounted displays were used. Patients used 2 different VR headsets depending on their age group (Samsung Gear VR [Samsung Electronics] for patients aged 13-21 years and Merge VR [Merge Labs] for patients aged 10-12 years). Both age groups played a multisensory (visual and auditory) VR game (Bear Blast; AppliedVR). While playing the game, users traveled on a preset path through a colorful highly interactive 3-dimensional environment filled with animated landscapes, buildings, and clouds, during which the user’s gaze controlled the direction of a cannon fired to knock down teddy bears. For hygiene purposes, patients wore hospital hairnets while using the VR equipment, and all equipment was cleaned with alcohol-based sanitary wipes between patients. The VR intervention was implemented by trained and supervised research staff (including A.S.L.). Over the course of the clinical trial, we did not receive any reports of misuse or mismanagement of the VR equipment.

### Measures

#### Demographic Characteristics

Patients 18 years and older self-reported their demographic information, including age, educational level, race/ethnicity, and medical history (eg, presence of chronic disease). For those younger than 18 years, a caregiver reported the patient’s age, grade, race/ethnicity, and medical history.

#### Pain

Patients and caregivers completed the Faces Pain Scale–Revised (FPS-R) to measure patient pain before and during the PIVC procedure.^28^ The FPS-R constitutes a horizontal series of 6 faces displaying a range of facial expressions, from no pain (0 points) to very much pain (10 points). Patients and caregivers were asked to point to the face that reflected the patient’s current level of pain. The FPS-R has been widely studied and found to have high reliability and validity in children aged 4 to 16 years.^29^ Clinicians also rated the patient’s level of pain during the procedure from 0 to 10 points.

#### Anxiety

Patients and caregivers completed a visual analogue scale (VAS) to measure patient anxiety before and during the PIVC procedure. The VAS for anxiety consisted of a vertical image of a thermometer that shifted in color from yellow (accompanied by an image of a neutral face) at the bottom to dark red (accompanied by an image of a distressed face) at the top. Patients and caregivers were asked to point to the spot on the thermometer that corresponded with the patient’s level of anxiety, with the neutral face scored as 0 points and the distressed face scored as 10 points. Visual analogue scales have been found to have good reliability and validity in pediatric samples.^30^ Clinicians also rated the patient’s level of anxiety during the procedure from 0 to 10 points.

#### Anxiety Sensitivity

Patients reported their anxiety sensitivity using the Childhood Anxiety Sensitivity Index (CASI).^31^ This 18-item measure uses a 3-point Likert scale to assess the extent to which respondents believe the experience of anxiety will result in negative consequences (with 1 indicating no negative consequences, 2 indicating some negative consequences, and 3 indicating a lot of negative consequences). Items were summed (score range, 18-54 points, with higher scores indicating higher levels of anxiety sensitivity). The CASI has been found to have high internal consistency (α = .87) and good test-retest reliability in both clinical (*r* = 0.79) and nonclinical (*r* = 0.76) samples.^31^

#### VR Immersion

After the PIVC procedure, patients in the VR group completed the Gold-Rizzo Immersion and Presence (GRIP) Inventory (eFigure in Supplement 2) to assess their degree of immersion in the game (with 1 indicating no immersion, 2 indicating a little immersion, and 3 indicating a lot of immersion). This 16-item measure was developed by an investigator (J.I.G.) and asks patients to respond to items in 3 domains: sense of involvement, perceived realism of the VR experience, and sense of transportation into the experience. Scores on the GRIP Inventory were summed (range, 0-32 points, with higher scores indicating higher levels of immersion) for patients in the VR group who completed at least 12 of the 16 items.

### Statistical Analysis

A power analysis using preliminary data from a previous study of VR use among patients receiving phlebotomy, which was conducted by the principal investigator (J.I.G.),^1^ determined that 100 patients were needed for this study. Recruitment stopped at 110 patients to account for dropout. Baseline characteristics were summarized using frequencies with percentages for categorical variables and medians with interquartile ranges (IQRs) for continuous variables. To determine differences in baseline characteristics between groups, χ^2^ or Fisher exact tests were used for categorical variables, and the Mann-Whitney U test was used for continuous variables.

The current analysis focused on the effectiveness of VR with regard to reducing pain and anxiety. Primary outcomes were post-PIVC anxiety and pain scores obtained from each of the 3 raters (comprising 6 of the 13 outcomes measured in the larger clinical trial). A secondary outcome was identification of patient factors within the VR group that were associated with better pain and anxiety outcomes. Generalized linear models were built to investigate differences between the patient groups using backward stepwise selection, allowing all variables to be entered and removed. Sex and location of care (categorized as radiology department vs infusion center) were the stratification variables; therefore, these variables were not eliminated from the models, whereas candidate variables, such as age (analyzed using continuous models and categorized as <14.7 years vs ≥14.7 years [based on median age] for presentation purposes only) and anxiety sensitivity (analyzed using continuous models and categorized as a CASI score of <8 points vs ≥8 points [based on median score] for presentation purposes only), were assessed for removal from and reentry in the models based on statistical significance. All models controlled for the patient’s pre-PIVC scores (analyzed using continuous models and categorized as a VAS score of <1.47 points vs ≥1.47 points [based on median score] and an FPS-R score of 0 points vs >0 points [based on median score] for presentation purposes only).

Within each outcome, separate models for each rater were constructed. The adjusted mean post-PIVC scores for the VR and standard care groups were presented with 95% CIs. Interactions between patient group and the presence of chronic disease at baseline were explored in the patient models for each outcome to determine whether chronic disease at baseline modulated the effectiveness of the VR intervention. Secondary analyses explored which patient characteristics within the VR group were associated with decreased pain and anxiety.

All *P* values were assessed at the 2-tailed .05 significance level. Data were analyzed using SAS software, version 9.4 for Windows (SAS Institute).

## Results

A total of 273 patients were assessed for eligibility. Of those, 102 patients (37.4%) declined participation, primarily because of disinterest in receiving the VR intervention or limited time to participate in the study (Figure). Overall, 118 patients were enrolled and randomized (60 patients in the standard care group and 58 patients in the VR group). Of those, 57 patients in the standard care group received intervention as randomized (the PIVC procedure was canceled for 2 patients, and 1 patient withdrew because of disappointment with standard care), and 53 patients in the VR group received intervention as randomized (the PIVC procedure was canceled for 2 patients, and 3 patients wanted to observe PIVC placement). After excluding 3 patients in the standard care group who received intervention in a location other than the radiology department or infusion center, a total of 107 patients (54 patients in the standard care group and 53 patients in the VR group) were included in the primary analysis. Each patient younger than 18 years was part of a triad that included 1 caregiver and 1 clinician; for patients 18 years and older, a caregiver was not included.

**Figure.**
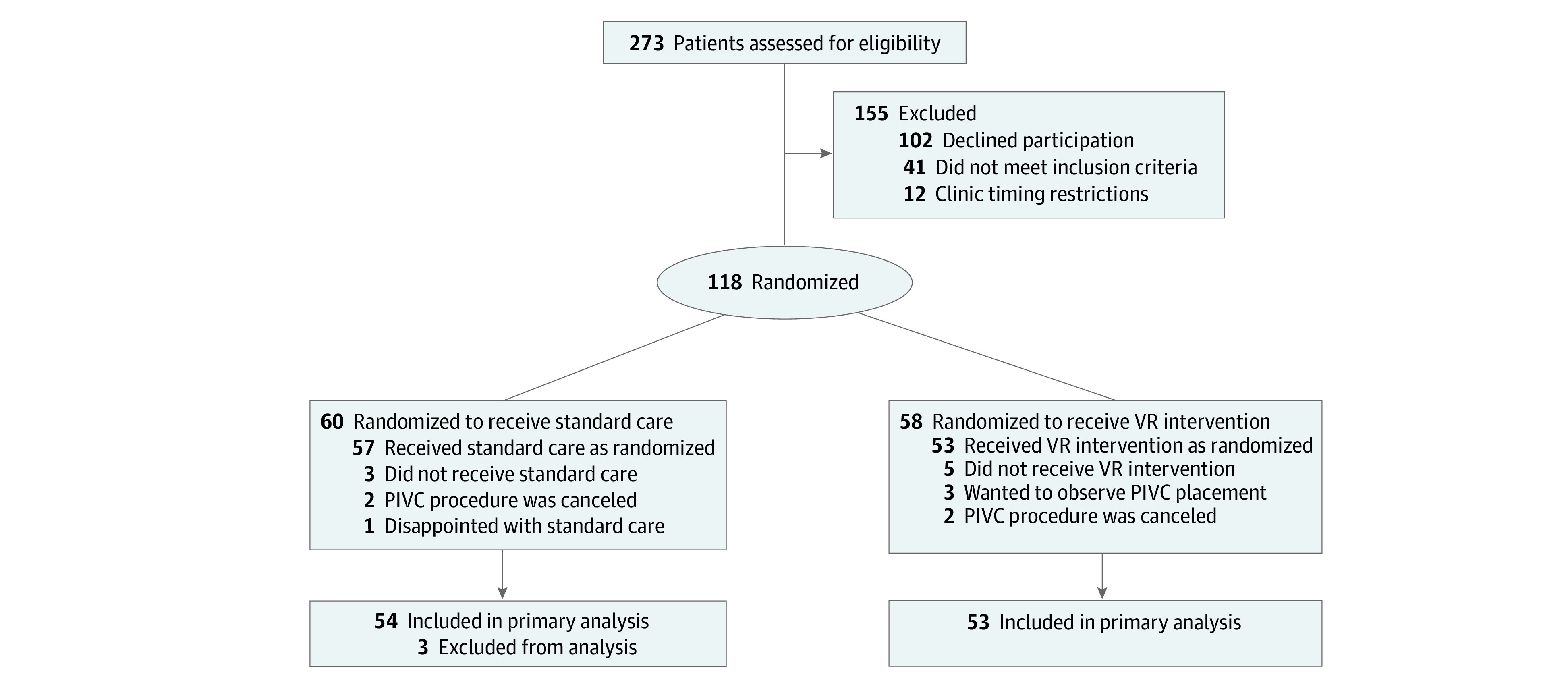
Study Flow Diagram PIVC indicates peripheral intravenous catheter; VR, virtual reality.

Among patients, the median age was 14.7 years (IQR, 12.8-16.9 years), and 63 patients (58.9%) were male (Table 1). In total, 86 of 101 patients (85.1%) had chronic disease at baseline. Among caregivers, most participants were mothers (64 women [59.8%]). The mean difference (using unequal variances) in post-PIVC anxiety (as measured by VAS score) between the standard care and VR groups was 1.51 points (95% CI, 0.67-2.35 points), and the mean difference (using pooled variances) in post-PIVC pain (as measured by FPS-R score) was 1.09 points (95% CI, 0.31-1.87 points) (Table 2).

**Table 1.** Baseline Patient Characteristics

Characteristic	No./total No. (%)		
	All	Standard care group	VR group
Total participants, No.	107	54	53
Sex			
Male	63/107 (58.9)	28/54 (51.9)	35/53 (66.0)
Female	44/107 (41.1)	26/54 (48.1)	18/53 (34.0)
Location of care			
Radiology department	42/107 (39.3)	21/54 (38.9)	21/53 (39.6)
Infusion center	65/107 (60.7)	33/54 (61.1)	32/53 (60.4)
Race/ethnicity			
Hispanic or Latino	46/106 (43.4)	22/53 (41.5)	24/53 (45.3)
White or non-Hispanic	22/106 (20.8)	12/53 (22.6)	10/53 (18.9)
Black or African American	10/106 (9.4)	7/53 (13.2)	3/53 (5.7)
Asian or Pacific Islander	8/106 (7.5)	5/53 (9.4)	3/53 (5.7)
Biracial	6/106 (5.7)	3/53 (5.7)	3/53 (5.7)
Multiracial	3/106 (2.8)	1/53 (1.9)	2/53 (3.8)
Other^a^	11/106 (10.4)	3/53 (5.7)	8/53 (15.1)
Age, median (IQR), y	14.7 (12.8-16.9)	14.9 (13.1-16.9)	14.4 (12.2-16.7)
Chronic disease	86/101 (85.1)	45/52 (86.5)	41/49 (83.7)
Pre-PIVC anxiety (VAS) score^b^			
Patients	105/107 (98.1)	54/54 (100)	51/53 (96.2)
Median (IQR)	1.47 (0.32-2.94)	1.56 (0.32-3.02)	1.32 (0.30-2.94)
Pre-PIVC pain (FPS-R) score^c^			
Patients	106/107 (99.1)	54/54 (100)	52/53 (98.1)
Median (IQR)	0 (0.00-0.00)	0 (0.00-0.00)	0 (0.00-0.00)
Anxiety sensitivity (CASI) summed score, median (IQR)^d^	8.00 (5.00-14.00)	8.50 (6.00-13.00)	8.00 (5.00-14.00)
VR immersion (GRIP Inventory) score^e^			
Patients	52/107 (48.6)	NA	52/53 (98.1)
Median (IQR)	24.00 (20.00-27.00)	NA	24.00 (20.00-27.00)

Abbreviations: CASI, Childhood Anxiety Sensitivity Index; FPS-R, Faces Pain Scale–Revised; GRIP, Gold Rizzo Immersion and Presence; IQR, interquartile range; NA, not applicable; PIVC, peripheral intravenous catheter; VAS, visual analogue scale; VR, virtual reality.

^a^ Races included in this category were not specified by participants.

^b^ Score range on the VAS: 0-10 points, with 0 indicating no anxiety and 10 indicating a high level of anxiety.

^c^ Score range on the FPS-R: 0-10 points, with 0 indicating no pain and 10 indicating very much pain.

^d^ Score range on the CASI: 18-54 points, with higher scores indicating higher levels of anxiety sensitivity.

^e^ Score range on the GRIP Inventory: 0-32, with higher scores indicating higher levels of VR immersion.

**Table 2.** Patients’ Pain and Anxiety Outcomes

Outcome	Patient post-PIVC score		Score difference between standard care and VR groups, mean (95% CI)
	Mean (SD)	Median (IQR)	
FPS-R pain score^a^			
All patients (n = 107)	1.64 (2.09)	2.00 (0-2.00)	
Standard (n = 54)	2.19 (2.21)	2.00 (0-2.00)	1.09 (0.31-1.87)^b^
VR (n = 53)	1.09 (1.82)	0 (0-2.00)	
VAS anxiety score^c^			
All patients (n = 106)	2.54 (2.31)	1.75 (0.68-3.96)	
Standard (n = 54)	3.28 (2.52)	2.63 (1.28-4.62)	1.51 (0.67-2.35)^d^
VR (n = 52)	1.78 (1.79)	0.98 (0.52-2.67)	

Abbreviations: FPS-R, Faces Pain Scale–Revised; IQR, interquartile range; PIVC, peripheral intravenous catheter; VAS, visual analogue scale; VR, virtual reality.

^a^ Score range on the FPS-R: 0-10 points, with 0 indicating no pain and 10 indicating very much pain.

^b^ Calculated using pooled variances.

^c^ Score range on the VAS: 0-10 points, with 0 indicating no anxiety and 10 indicating a high level of anxiety.

^d^ Calculated using unequal variances.

### Anxiety

Multiple linear regression analyses indicated that patients in the VR group had significantly lower adjusted mean post-PIVC anxiety scores on the VAS compared with patients in the standard care group when anxiety was patient-reported (1.85 points [95% CI, 1.28-2.41 points] vs 3.14 points [95% CI, 2.59-3.68 points]; *P* < .001) and clinician-reported (2.04 points [95% CI, 1.37-2.71 points] vs 3.34 points [95% CI, 2.69-3.99 points]; *P* = .002). There was no evidence that chronic disease at baseline modulated the effectiveness of the VR intervention; therefore, the interaction between chronic disease and patient group was not included in the model.

No significant difference was found in the VR vs standard care groups when the patient’s anxiety was caregiver-reported (2.62 points [95% CI, 1.72-3.52 points] vs 3.19 points [95% CI, 2.34-4.04 points]; *P* = .32). Older patient age (≥14.7 years vs <14.7 years) was associated with lower anxiety scores when rated by the clinician (2.11 points [95% CI, 1.45-2.78 points] vs 3.27 points [95% CI, 2.61-3.93 points]; *P* = .01) but not when rated by the patient or caregiver (Table 3).

**Table 3.** Results From Generalized Linear Models Across Raters^a^

Variable	Post-PIVC anxiety (VAS) or pain (FPS-R) score^b^					
	Patients		Caregivers		Clinicians	
	Adjusted mean (95% CI)^c^	*P* value^d^	Adjusted mean (95% CI)^c^	*P* value^d^	Adjusted mean (95% CI)^c^	*P* value^d^
Anxiety^e^						
Patient pre-PIVC anxiety (VAS) score						
<1.47	1.47 (0.91-2.04)	<.001	2.23 (1.30-3.16)	.01	2.17 (1.49-2.84)	.002
≥1.47	3.51 (2.96-4.06)		3.58 (2.74-4.42)		3.22 (2.57-3.87)	
Location of care						
Radiology department	2.35 (1.73-2.97)	.34	3.11 (2.17-4.05)	.50	2.43 (1.71-3.16)	.21
Infusion center	2.63 (2.14-3.12)		2.70 (1.88-3.52)		2.95 (2.35-3.55)	
Patient sex						
Female	2.44 (1.83-3.05)	.93	3.31 (2.36-4.26)	.14	2.88 (2.16-3.61)	.34
Male	2.54 (2.03-3.05)		2.50 (1.69-3.31)		2.50 (1.89-3.11)	
Patient group						
VR	1.85 (1.28-2.41)	<.001	2.62 (1.72-3.52)	.32	2.04 (1.37-2.71)	.002
Standard care	3.14 (2.59-3.68)		3.19 (2.34-4.04)		3.34 (2.69-3.99)	
Patient age, y						
<14.7	NA	NA	NA	NA	3.27 (2.61-3.93)	.01
≥14.7	NA		NA		2.11 (1.45-2.78)	
Pain^f^						
Patient pre-PIVC pain (FPS-R) score						
0	1.42 (0.97-1.86)	.09	2.14 (1.58-2.69)	.44	2.33 (1.95-2.71)	.02
>0	2.46 (1.32-3.60)		2.74 (1.22-4.26)		3.32 (2.40-4.24)	
Location of care						
Radiology department	1.83 (1.05-2.62)	.65	2.30 (1.33-3.28)	.65	2.66 (2.02-3.29)	.41
Infusion center	2.04 (1.36-2.73)		2.57 (1.64-3.51)		2.99 (2.42-3.56)	
Patient sex						
Female	1.80 (1.05-2.54)	.43	2.55 (1.57-3.53)	.61	2.97 (2.35-3.58)	.31
Male	2.08 (1.35-2.81)		2.32 (1.39-3.25)		2.68 (2.08-3.28)	
Patient group						
VR	1.34 (0.63-2.05)	.002	1.87 (0.99-2.76)	.04	2.05 (1.47-2.63)	<.001
Standard care	2.54 (1.78-3.30)		3.01 (1.98-4.03)		3.59 (2.97-4.22)	
Patient age, y						
<14.7	2.20 (1.48-2.93)	.03	NA	NA	NA	NA
≥14.7	1.67 (0.92-2.42)		NA		NA	
Patient anxiety sensitivity (CASI) summed score^g^						
<8	1.73 (0.87-2.59)	.01	NA	NA	NA	NA
≥8	2.15 (1.53-2.77)		NA		NA	

Abbreviations: CASI, Childhood Anxiety Sensitivity Index; FPS-R, Faces Pain Scale–Revised; NA, not applicable (variable removed from model because it was not statistically significant); PIVC, peripheral intravenous catheter; VAS, visual analogue scale; VR, virtual reality.

^a^ Patient pre-PIVC scores, location of care, sex, and patient group were included in the model. Backward stepwise selection with a significance threshold of *P* < .05 was used to determine whether age and patient CASI summed score would remain in the final model.

^b^ Pre- and post-PIVC anxiety was measured by VAS score (range, 0-10 points, with 0 indicating no anxiety and 10 indicating a high level of anxiety), and pre- and post-PIVC pain was measured by FPS-R score (range, 0-10 points, with 0 indicating no pain and 10 indicating very much pain).

^c^ Adjusted means are the estimated mean outcome scores adjusted for all other variables in the model. Adjusted means for continuous variables were split at the median value for reporting purposes.

^d^ *P* values are based on the final model containing the continuous version of continuous variables (if included in the final model).

^e^ Includes 105 patients, 84 caregivers, and 101 clinicians.

^f^ Includes 106 patients, 88 caregivers, and 102 clinicians.

^g^ Score range on the CASI: 18-54 points, with higher scores indicating higher levels of anxiety sensitivity.

### Pain

After adjusting for other variables in the multivariable model, patients in the VR group had significantly lower adjusted mean post-PIVC pain scores on the FPS-R compared with those in the standard care group when pain was patient-reported (1.34 points [95% CI, 0.63-2.05 points] vs 2.54 points [95% CI, 1.78-3.30 points]; *P* = .002), caregiver-reported (1.87 points [95% CI, 0.99-2.76 points] vs 3.01 points [95% CI. 1.98-4.03 points]; *P* = .04), and clinician-reported (2.05 points [95% CI, 1.47-2.63 points] vs 3.59 points [95% CI, 2.97-4.22 points]; *P* < .001). In the patient model, the interaction between chronic disease at baseline and patient group was removed from the model because there was no evidence that chronic disease modulated the effect of the VR intervention on patient post-PIVC anxiety. In the same model, older patient age (≥14.7 years vs <14.7 years) was associated with lower mean pain scores (1.67 points [95% CI, 0.92-2.42 points] vs 2.20 points [95% CI, 1.48-2.93 points]; *P* = .03), whereas higher patient CASI summed scores (≥8 points vs <8 points) were associated with higher mean pain scores (2.15 points [95% CI, 1.53-2.77 points] vs 1.73 points [95% CI, 0.87-2.59 points]; *P* = .01), regardless of treatment group (Table 3).

### Patients Who Benefited From VR Intervention

A significant positive association was observed between pre-PIVC pain and anxiety scores vs post-PIVC pain and anxiety scores among patients in the VR group. When the patient’s pre-PIVC pain score on the FPS-R was 0, the adjusted mean post-PIVC pain score was 0.88 points (95% CI, 0.34-1.43 points) vs 2.22 points (95% CI, 1.03-3.41 points; *P* = .045) when the pre-PIVC pain score was greater than 0. When the patient’s pre-PIVC VAS anxiety score was less than 1.47, the adjusted mean post-PIVC anxiety VAS score was 1.15 points (95% CI 1.15-1.78) compared with those with a pre-PIVC pain score of 1.47 or greater, who had an adjusted mean post-PIVC anxiety score of 2.58 points (95% CI 1.87-3.28). In the VR group, age, location of care, sex, VR immersion (GRIP Inventory) score, and patient anxiety sensitivity (CASI) summed score were not associated with changes in outcomes (Table 4).

**Table 4.** Results from Linear Modeling Within the Virtual Reality Group^a^

Variable	Post-PIVC score, adjusted mean (95% CI)^b^	*P* value^c^
Patient pre-PIVC anxiety (VAS) score (n = 51)^d^		
<1.47	1.15 (0.51-1.78)	.008
≥1.47	2.58 (1.87-3.28)	
Patient pre-PIVC pain (FPS-R) score (n = 52)^e^		
0	0.88 (0.34-1.43)	.045
>0	2.22 (1.03-3.41)	

Abbreviations: CASI, Childhood Anxiety Sensitivity Index; FPS-R, Faces Pain Scale–Revised; PIVC, peripheral intravenous catheter; VAS, visual analogue scale.

^a^ Models were built using backward stepwise selection starting with possible candidate independent variables comprising patient pre-PIVC scores, location of care, sex, age, VR immersion score, and CASI summed score.

^b^ Adjusted means are the estimated mean outcome scores adjusted for all other variables in the model. Adjusted means for continuous variables were split at the median value for reporting purposes.

^c^ *P* values are based on the final model containing the continuous version of continuous variables (if included in the final model).

^d^ Score range on the VAS: 0-10 points, with 0 indicating no anxiety and 10 indicating a high level of anxiety.

^e^ Score range on the FPS-R: 0-10 points, with 0 indicating no pain and 10 indicating very much pain.

## Discussion

In this randomized clinical trial, the hypothesis that a VR intervention compared with standard care would decrease pain and anxiety among patients undergoing PIVC placement was supported by the findings. In addition, the question of which patients benefit most from VR intervention was partially answered; among several independent variables (including location of care, sex, age, VR immersion score on the GRIP Inventory, presence of chronic disease at baseline, and patient anxiety sensitivity score on the CASI), only pre-PIVC pain and pre-PIVC anxiety scores were associated with changes in post-PIVC pain and post-PIVC anxiety scores for patients who received the VR intervention. Investigators have previously raised the question of whether young patients who have chronic health conditions and undergo more frequent PIVC procedures respond differently to VR intervention.^1^ In the current study, the VR intervention was effective in reducing pain and anxiety, irrespective of the patient’s chronic disease status.

This study’s findings add to the evidence-based literature indicating that a VR intervention is capable of decreasing pain and anxiety among patients undergoing painful procedures. In addition to quantitative decreases in pain and anxiety, the qualitative reports of patient, caregiver, and clinician satisfaction obtained in this study may have meaningful clinical implications. A previous study found that receipt of painful medical procedures was associated with a person’s overall perceptions of adverse childhood experiences, which in turn has been reported to have negative implications for long-term health outcomes.^32^ Few VR studies have conducted prospective randomized clinical trials with a large cohort.^33,34^ This study is the first, to our knowledge, to prospectively investigate a large cohort across 2 clinical settings (a radiology department and an infusion center). The study’s results support the effectiveness of VR in both settings, with no significant differences between settings found, and they extend the findings reported in a 2018 study of VR use among patients receiving phlebotomy.^1^ Scientific investigation of VR interventions across a variety of settings is necessary and may lend support for the flexibility of VR systems as nonpharmacological interventions for pain management.

This clinical trial conducted a series of distinct analyses, triangulating the data around the patient using patient-reported, caregiver-reported, and clinician-reported scores for pain and anxiety. To our knowledge, these analyses have never been conducted in VR studies and reflect the discrete contributions of patient, caregiver, and clinician perspectives in assessing patient pain and anxiety. The current data suggest that the significance and extent of the effects of patient age, sex, location of PIVC procedure, VR intervention, and patient anxiety sensitivity differed among the 3 ratings of pain and anxiety. The VR intervention was beneficial in reducing pain in all 3 rating models and reducing perceived anxiety in 2 rating models (patient and clinician); however, perceived anxiety was not significantly different when reported by the caregiver. Future research would benefit from continuing to examine multirater assessments, including ratings from patients, caregivers, and clinicians. Additional objective measurement of outcomes, including video recordings of the patient experience before, during, and after medical procedures, would provide valuable data.

The data from this study support the management of pain and anxiety using a VR intervention among patients undergoing PIVC needle-related procedures in the pediatric setting and highlight the opportunity for nonpharmacological interventions. Given the ongoing opioid crisis, there is substantial concern about the role of medications in pain management.^35^ Virtual reality, a nonpharmacological intervention, is an empirically supported, feasible, and cost-effective solution to managing pain and anxiety during routine venipuncture procedures in the pediatric setting. Virtual reality interventions can target the reduction of needle phobia; reduce adverse and traumatic reactions to medical procedures; improve satisfaction for patients, caregivers, and health care practitioners; and lead to improved outcomes as a result of consistent adherence to treatment. Future research on reducing pain and anxiety during medical procedures (eg, magnetic resonance imaging, PIVC placement, lumbar puncture, otolaryngologic procedures, and cast removal) may benefit from examining interventions that reduce or eliminate the use of medications (ie, anxiolytic and narcotic drugs). A movement toward nonsedating medical procedures using VR interventions may reduce many of the known adverse effects of medications and improve overall health outcomes for patients with acute and/or chronic medical needs.

### Limitations

This study has limitations. The current findings support the use of a VR intervention for PIVC procedures in the pediatric setting; however, a number of issues should be considered when implementing VR within both inpatient and outpatient clinics. Although this study used a prospective randomized clinical trial design, the nature of patient-reported, caregiver-reported, and clinician-reported outcomes introduces bias and subjectivity. Because this study was unblinded and collected self-reported data, the patients, caregivers, and clinicians may have been motivated by a desire to please the investigator when completing the measures. These findings are also limited to the performance of a PIVC procedure in 1 radiology department and 1 outpatient infusion center at a single pediatric academic hospital. Although future studies would benefit from examining the impact of combined treatments (ie, VR plus standard care), the size of the current sample did not permit this type of analysis.

The VR hardware and software were consistently used across both clinical environments in this study, which established a sound and rigorous methodological approach; however, this approach limits the potential generalizability of the findings to 2 VR systems (ie, Samsung Gear VR powered by Oculus and the Merge VR headsets) and 1 VR experience (ie, Bear Blast) at 1 pediatric medical center. Although the field of digital therapeutics has lacked scientific data to understand which VR systems are best suited for specific interventions, the findings of the current study reflect the increasing body of evidence supporting the benefits of specific VR systems (eg, Bear Blast) for the management of pain and anxiety associated with both blood sample collection and PIVC placement.^1,36,37^ Given the rapidly evolving technological development of both VR hardware and software, the validation of a single VR system has inherent strengths and limitations. Although it is becoming increasingly feasible to integrate VR systems into clinical practice because of the reduced costs, availability of high-quality off-the-shelf technologies, and large number of VR environments, requirements remain regarding training and supervision of staff to operate the VR equipment, strict and vigilant hygiene practices based on hospital infectious disease standards, powering the equipment, and performance of routine updates on the operating systems and software. Misuse or mismanagement of the VR equipment could result in ineffective and problematic use.

Although positive results have been reported regarding VR interventions for the management of acute procedural pain over the past 20 years, the costs, availability of hardware and software, and lack of resources to execute these interventions have hindered widespread use.^1,38^ As VR systems continue to become more affordable, child life programs and other stakeholders may benefit from the introduction of a VR intervention into routine blood sample collection and PIVC procedures. The use of VR systems for reducing pain and anxiety during acute painful medical procedures, especially blood sample collection and PIVC placement, continues to be supported in the literature, from single case studies to larger-scale randomized clinical trials.^1,39,40,41,42,43^

Combining technological innovation with scientific rigor to systematically evaluate the use of VR interventions to solve difficult health care issues has been challenging, but successful integration of these 2 areas could substantially improve nonpharmacological interventions for pain and procedural anxiety and/or distress. Many academic and private organizations are focused on developing hardware and software for VR and other digital therapeutics for implementation in health care settings.^44,45^ Although the current study focused on the use of a VR intervention for managing acute pain and anxiety associated with PIVC placement, other researchers are focusing on virtual therapeutics as a solution for a number of health care problems. Harnessing the innovation and accessibility of VR to deliver nonpharmacological interventions for routine painful medical procedures may have substantial physiological and mental health implications.

## Conclusions

The results of this randomized clinical trial are statistically and clinically significant and support the role of VR systems during PIVC procedures in 2 pediatric clinical settings. A VR intervention significantly decreased pain and anxiety among patients undergoing PIVC placement in the pediatric setting.

## Notes

Supplement 1.
Trial Protocol

Supplement 2. **eFigure.** Gold-Rizzo Immersion and Presence (GRIP) Inventory

Supplement 3.
Data Sharing Statement
