# Supplementary material for: Effect of an Immersive Virtual Reality Intervention on Pain and Anxiety Associated With Peripheral Intravenous Catheter Placement in the Pediatric Setting: A Randomized Clinical Trial
Source: JAMA Netw Open. 2021 Aug 25;4(8):e2122569. doi: 10.1001/jamanetworkopen.2021.22569 (PMC8387848; doi:10.1001/jamanetworkopen.2021.22569)
Supplement: Supplement 1. — Trial Protocol [file jamanetwopen-e2122569-s001.pdf]

USC

Date: Friday, August 28, 2020 4:40:24 PM

Print Close

iStar ID: CHLA-15-00549

Application Version Date:7/21/2020 Version:19.0

CHLA-15-00549

View: 12. Methods and Procedures - Prospective Studies

12. Methods and Procedures - Prospective Studies

12.1. Describe in detail the design and methodology of the study. Provide a detailed description of the planned data collection, specific outcomes, and criteria for evaluation and endpoint definition. If applicable, include information on stratification or randomization plans. Include the frequency and duration of each activity and the total length of subject participation. Identify and distinguish between those procedures that are standard of care and those that are experimental. (***Refer to specific sections of the protocol/grant, if applicable. Describe any differences between the protocol and the local site.***)

A stratified and randomized control trial will be conducted to examine the specific aims of the proposed study. Each eligible patient will be randomized to one of two groups, standard of care or standard of care plus virtual reality (VR). A balanced randomization scheme will be used to balance the age and gender distribution of the two study groups.

Stratified Randomization Design
Contingent on randomization, the patient will undergo the medical procedure either according to CHLA's standard of care (control) or the patient will participate in the VR plus standard of care (VR) condition. In order to balance the number of patients by gender, we will use a stratified randomization scheme for assignment of patients into study groups. Gender will be divided into two strata. Within each gender stratum, a balanced randomization scheme will be generated to assure equal number of VR and standard of care subjects. At the beginning of the project, the investigative team will develop the randomization schema within SPSS 22.0 that will be used for the proposed recruitment design. For implementation, the investigative team will refer to the list generated by the randomization program to determine which condition the next study subject will be assigned to. This list will be divided into the two gender groups described above.

Experimental Conditions

This study will compare the subjective reports of children, caregivers, and healthcare providers who participate in a randomized crossover trial of VR versus standard of care (control). In the standard of care treatment condition, participants will receive the standard CHLA treatment protocol for the medical procedure. The intervention group will receive standard CHLA treatment with VR distraction.

Children and their caregivers will informed that they will be randomized to either the VR or standard of care condition. They will be told about both conditions and informed that if they are assigned to the control condition, patients will be able to play with the VR after the medial procedures and study measures are completed. Children will be told that they cannot decide on their treatment condition. Children in the VR condition will undergo the invasive procedure while distracted by interaction with an immersive virtual environment (VE) presented via a head mounted display (HMD).

Virtual Reality Technology

Four types of VR HMDs are available for use in this study. Participants 13 -21 years old can use the Samsung Gear VR. Participants 10-21 years can use the Merge, Oculus Go, and Mira Prism. The VE to be used in this study is mobile based and has active matrix LCDs with high pixel resolution, creating a bright, vibrant color and a quality image. This VE has already been utilized in lab studies with children without any reported or observed difficulties.

Within the VE, users can watch age-appropriate movies (up to parent's discretion) or engage in BearBlast (developed by Applied VR). The movie-watching VE places the user in a virtual movie theater environment, complete with velvet red seats. No movement is required and the user only has to look at the large movie screen ahead to watch the movie. In BearBlast, users travel on a pre-set path through a colorful, vibrant, highly interactive environment, filled with toy-like trees, mountains, rainbows, mushrooms, and bushes. As they gaze, they control the direction of a continuously firing cannon, which interacts with items in the world and positively reinforces experimentation and activity. They automatically enter a new level every 2.5 minutes, keeping the environment continuously fresh. Throughout the world, plush moles play the role of antagonist, standing in for the nuisance and distraction of pain. The VR game is equipped with a head-tracking system, enabling the player to look around the virtual environment. In addition, there is the option to interact with the VR environment using a tap pad located on the side of the helmet. Therefore, the child will be receiving distraction via 3-D visual and auditory sensory, and tactile feedback, thus supplying a multi-sensory immersive experience. While wearing these glasses, the children only can see the HMD screen so that the immersion and presence will be increased. The VR glasses will be sanitized before and after every use so that the chance of infection will be minimized.

The Mira Prism is a portable, augmented reality (AR) HMD powered by iPhone. When paired with the iPhone, the Mira Prism goggles can superimpose computer-generated images on the user's view of the real world. Unlike the full immersion of VR, AR allows users to view the outside world while interacting with digital content. In this study, the patient can interact with Magic Mallet (developed by Miney Moe). Magic Mallet is a distraction game designed for pain management and communication during procedures. The game automatically adjusts cognitive load for optimal effect for users. Study team iPhones specifically loaded with Magic Mallet, and without cellular capability, will be used with the Mira Prism.

We will ensure that the Mira Prism is not used with patients who have symptoms of "squint, amblyopia, or anisometropia" through our eligibility screening of all patients, performed prior to the informed consent process. One of the exclusion criteria in our study is "Children with visual, auditory, or tactile deficits that would interfere with the ability to complete the experimental tasks or use the technological devices." We will not enroll children with any visual deficits.

The manuals for Oculus Go and Mira Prism both state that these devices should not be used by children under 13 years of age because the headset is not sized for children and improper sizing can lead to discomfort. This warning has to do with hardware design rather than actual safety. Both devices have been used across the country by children under 13 years of age. We will always tighten the adjustable straps of these devices to ensure that they are comfortable for children under 13 years.

Patient Procedures and Data Collection

After informed consent and assent have been obtained and the patient has successfully met the screening criteria for cognitive ability, the patient and his/her caregiver will be enrolled into the study. Patients and their caregivers will be asked to fill out several questionnaires via Qualtrics on an individual IPAD (see uploaded Table of Measures) in the private area/room.

In order to ensure that pre-existing pain or anxiety do not contaminate reports of procedural pain or virtual reality-related symptomatology, the patient will be asked to report pre-procedure baseline levels for the following measures: the Visual Analog Scale (VAS), the Faces Pain Scale Revised (FPS-R), the Colored Analogue Scale (CAS) for pain, the VAS for procedural anxiety, the Facial Affective Scale (FAS), the Childhood Anxiety Sensitivity Index (CASI), and the Malaise Scale. If patient is age 18 or above, they will also complete a demographic form. This is expected to take approximately 15 minutes.

After patients have completed the pre-procedural battery on the IPAD, and patients and caregivers will be informed about their assigned study condition (VR or standard of care). Children in the VR distraction groups will then begin interacting with the virtual environment for approximately 5 minutes. Following the 5-minute VR primer period the procedure will commence. Following the procedure, children will continue to play the VR game for approximately 2 minutes. Children in the standard care control group will undergo the medical procedure according to the existing CHLA protocol. Irregardless of treatment condition, the Research Assistant will observe and record the behavior of the child while s/he is undergoing the medical procedure. A video tape recording of the procedure will also be obtained (if the child, caregiver, and healthcare provider have agreed to be recorded) in order to later score the behavior of the child according to the Child-Adult Medical Procedure Interaction Scale – Revised (CAMPIS-R). Video will be focused on the patient/caregiver and not on the healthcare provider. However, it is possible that the healthcare provider's voice or appendage will be recorded while interacting with the patient and therefore their verbal consent will be obtained as well. Video recording will later be coded for signs and symptoms of anxiety and behavioral distress in the patient and his/her caregiver.

Following completion of the medical procedure, all children will complete the VAS and CAS to report pain intensity, the FPS-R, the VAS and FAS for procedural anxiety, the Malaise Scale, the Child Presence Questionnaire (VR group only), and the Satisfaction Questionnaire via Qualitrics on the IPAD. This is expected to require approximately 15 minutes.

Following completion of these measures, children who were not randomized to the VR condition will have the opportunity to use the VR.

Caregiver Procedures and Data Collection

After informed caregiver consent and child assent is obtained the caregiver will complete a brief demographic survey and baseline VAS, FPS-R, CAS, and FAS estimates of their child's pre-procedural pain and anxiety via Qualitrics on an individual IPAD. This will require approximately 10 minutes. Note that the caregiver may not participate if the patient is age 18 or above.

Following the medical procedure, caregivers of all children age 10-18 will complete VAS, FPS-R, and CAS measures to estimate their perception of their child's pain during the medical procedure and VAS and FAS measures of procedural anxiety. Parents will also complete a Satisfaction Questionnaire. These questionnaires will again be presented via Qualtrics on an individual IPAD. This will require approximately 10 minutes.

Healthcare Providers' Responsibilities and Involvement:

Healthcare providers will be asked to continue with their standard duties and to complete a VAS and CAS measure of their perceptions of the child's pain and anxiety via a healthcare provider survey (satisfaction questionnaire). Because the questionnaire is not part of their routine duties, they will be provided with an information form detailing the study procedures. Following completion of the medical procedure, the healthcare provider will complete a 7-item measure about their perception of the patient's cooperation and distress as well as their satisfaction with the procedure. Completion of this measure is expected to require approximately 5 minutes or less. If the healthcare provider does not wish to participate, the patient may still participate in the study, but will not be videotaped. The healthcare provider will not be asked to complete the questionnaire.

Study Team Procedures and Data Collection

The PI, Co-I, or research assistant will complete the case report form on the IPAD (before the child completes the post-measures). They will also complete observational measures (i.e., the CAMPIS-R) to record the patient's distress, modes of coping prior to the procedure, and other observational data during the medical procedure. With the consent of participants, all procedures will be videotaped, to allow a member of the study team to review the tape for more accurate behavioral observations. The video camera will focus on the patient's behavioral distress and reactions prior to and during the medical procedure. Video recording will focus on the participants rather than on the medical procedure. Video tapes will later be rated and coded based on the Child-Adult Medical Procedure Interaction Scale - Revised (CAMPIS-R) by two independent raters. An examination of inter-rater reliability (Kappa statistic) will be employed to evaluate the reliability between the coders and a accurate measure of the observational behavioral distress ratings.

Non-Randomization Protocol

There is a second version of the current protocol that does not include randomization for patients undergoing select painful procedures. In this version, all study subjects will use a virtual reality headset during the duration of the procedure. All participants will answer questions regarding the efficacy of VR similar to those answered by the experimental group participants in the original version of the current protocol.

12.2. Describe the statistical considerations for the study, how the sample size was determined, and how the results will be analyzed, if applicable. (***Refer to specific sections of the protocol/grant, if applicable***)

Power analyses based on preliminary data from the PI's previous VR and phlebotomy study determined that 100 participants are needed for the current study. The power calculation is based on the pain intensity VAS, which is calculated as a continuous (0 to 100) variable. 120 patients will be recruited for each type of painful medical procedure to account for attrition/drop out. In other words, 120 patients receiving venipuncture will be recruited, and a separate 120 patients receiving IV placement will be recruited. A total sample size of 100 patients per medical procedure type would result in 25 patients in each of the 4 age-gender strata groups. In our earlier preliminary data the VR pain score was about half that of the other methods giving an effect size of about 1.5. This would result in 80% power for a 2-sided t-test at the .05 significance level. Because we have several covariates planned in this study, we will increase the required sample size by (at least) 20% to allow for adjustment of the covariates. A general rule of thumb is to increase the sample size by 10-20% for each major covariate. Therefore, the proposed sample size of 240 (120 per procedure), assuming some attrition/drop-out will result in more children in each group.

Statistical Analysis

All data collected will be entered, coded, scored and analyzed using SPSS 22.0. Descriptive analyses will be performed to report demographics and to examine all means/standard deviations/correlations amongst the variables of interest. Once the integrity of the data is validated the following analyses will be conducted.

Aim 1. To determine if VR is more effective than standard care for reducing pain and anxiety in children undergoing painful medical procedures.

Hypothesis 1: Patients in the VR condition will report significantly less pain, anxiety, and distress (i.e., measured via self-report, parent-report, and behavioral observations compared to standard of care for patients undergoing a painful medical procedure.

For this hypothesis, there are 6 child outcome areas that will be evaluated: pain intensity, affective pain, child anxiety, anticipatory anxiety, and behavioral distress. When applicable, differences controlling for baseline variables, as well as differences in post measures will be evaluated. A univariate analysis will be performed to assess for differences in baseline variables between the two groups (VR versus standard of care). In addition the influence of any possible covariates such as child age, gender, or other demographic variables (e.g., previous experience with that medical procedure) might have on the six principal outcome measures will be investigated through a multivariate correlation analysis. If covariates are found to significantly relate to outcomes, they will be included in the multivariate analyses.

Aim 2. To evaluate caregiver's assessment patient distress and satisfaction with VR compared to standard of care for patients undergoing painful medical procedures.

Hypothesis 2: Caregivers will perceive less pain/anxiety in their children and greater have satisfaction during the VR condition compared to standard of care for patients undergoing painful medical procedures.

For this hypothesis, caregiver report of child pain, caregiver report of child anxiety, and caregiver satisfaction will be compared between the VR and the standard of care groups using a univariate analysis. The independent variable will be treatment type (standard of care/VR), and the dependent variables will be caregiver perception of child pain/anxiety, and caregiver satisfaction.

Aim 3: To evaluate healthcare provider's assessment of patient cooperation and their satisfaction with VR compared to standard of care.
Hypothesis 3: Healthcare providers will report greater patient cooperation and greater satisfaction in the VR condition compared to standard of care for patients undergoing a painful medical procedure.
For this hypothesis, healthcare provider's measurement of child cooperation and satisfaction will be compared between the VR and the standard of care groups using a univariate analysis. The independent variable will be treatment type (VR/standard of care), and the dependent variables will be child cooperation and healthcare provider satisfaction.
