# Supplementary material for: Effect of an Immersive Virtual Reality Intervention on Pain and Anxiety Associated With Peripheral Intravenous Catheter Placement in the Pediatric Setting: A Randomized Clinical Trial
Source: JAMA Netw Open. 2021 Aug 25;4(8):e2122569. doi: 10.1001/jamanetworkopen.2021.22569 (PMC8387848; doi:10.1001/jamanetworkopen.2021.22569)
Supplement: Supplement 2. — eFigure. Gold-Rizzo Immersion and Presence (GRIP) Inventory [file jamanetwopen-e2122569-s002.pdf]

Supplementary Online Content

Gold JI, SooHoo M, Laikin AM, Lane AS, Klein MJ. Effect of an immersive virtual reality intervention on pain and anxiety associated with peripheral intravenous catheter placement in the pediatric setting: a randomized clinical trial. *JAMA Netw Open*. 2021;4(8):e2122569. doi:10.1001/jamanetworkopen.2021.22569

eFigure. Gold-Rizzo Immersion and Presence (GRIP) Inventory

This supplementary material has been provided by the authors to give readers additional information about their work.

© 2021 Gold JI et al. *JAMA Network Open*.

eFigure. Gold-Rizzo Immersion and Presence (GRIP) Inventory

Gold-Rizzo Immersion and Presence (GRIP) Inventory

Study ID______________ Gender________ Age________ Date______________

Instructions: I am going to ask you some questions and I would like you to tell me how you felt **while you were playing the game.**

1. Did you enjoy the game?	No	A little	A lot
2. Did the game grab your attention?	No	A little	A lot
3. Did it feel like you were really there?	No	A little	A lot
4. Were you interested and involved in the game?	No	A little	A lot
5. Were you sad or disappointed when the game was over?	No	A little	A lot
6. Did you find the game easy to play?	No	A little	A lot
7. Were you worried about putting on the headset?	No	A little	A lot
8. Was the headset comfortable when you had it on?	No	A little	A lot
9. Were the controls easy to use?	No	A little	A lot
10. Did you get used to playing the game quickly?	No	A little	A lot
11. Did the things you saw look real?	No	A little	A lot
12. Did the way things moved look real?	No	A little	A lot
13. Did the things you heard sound real?	No	A little	A lot
14. Did it feel like you were in control of what happened in the game?	No	A little	A lot
15. Was the game interesting compared to other computer games you've played?	No	A little	A lot
16. Would you like to play the game again?	No	A little	A lot

© 2021 Gold JI et al. *JAMA Network Open*.

Item Scoring

No = 0
A little = 1
A lot = 2

Items adapted from following domains to enhance content validity and predict factors

**Domain**	**Item Number**
Involvement/ Immersion	2
	4
	10
	14
Realism	11
	12
	13
Transportation	3
Feasibility	6
	7*
	8
	9
Statisfaction	1
	5*
	15
	16

*item was reverse scored when summarizing composite scores

Item Customization

Researchers may customize items and instructions to tailor the measure to specific programs and games.

For Example:
Original: Did you feel like you were in control of what happened in the game?
Edit 1: Did you feel like you were in control of what happened in the *virutal reality* game?
Edit 2: Did you feel like you were in control of what happened in the *Bear Blast* game?

© 2021 Gold JI et al. *JAMA Network Open*.
