# Supplementary material for: Effect of an Immersive Virtual Reality Intervention on Pain and Anxiety Associated With Peripheral Intravenous Catheter Placement in the Pediatric Setting: A Randomized Clinical Trial
Source: JAMA Netw Open. 2021 Aug 25;4(8):e2122569. doi: 10.1001/jamanetworkopen.2021.22569 (PMC8387848; doi:10.1001/jamanetworkopen.2021.22569)
Supplement: Supplement 3. — Data Sharing Statement [file jamanetwopen-e2122569-s003.pdf]

Data Sharing Statement

Gold. Effect of an Immersive Virtual Reality Intervention on Pain and Anxiety Associated With Peripheral Intravenous Catheter Placement in the Pediatric Setting. *JAMA Netw Open*. Published August 25, 2021. doi:10.1001/jamanetworkopen.2021.22569

Data
Data available: Yes
Data types: Deidentified participant data
How to access data: jgold@chla.usc.edu
When available: With publication

Supporting Documents
Document types: None

Additional Information
Who can access the data: Individuals requested the data with a approved proposals to use the data.
Types of analyses: meta-analyses
Mechanisms of data availability: Following the approval of a proposal and signed data agreement for access.
Any additional restrictions: none
